# Preoperative assessment of NIFTP clinicopathological characteristics and its impact on avoiding overtreatment

**DOI:** 10.1007/s00423-025-03788-4

**Published:** 2025-07-01

**Authors:** M. Andrade de Almeida, P. Canão, J. Capela, P. Sá Couto, S. Carneiro

**Affiliations:** 1 Faculty of Medicine, University of Porto, Porto, Portugal; 2 General Surgery Department, São João Local Health Unit, Porto, Portugal; 3 Pathology Department, São João Local Health Unit, Porto, Portugal

**Keywords:** NIFTP, Papillary thyroid cancer, Premalignant, TBSRTC, EU-TIRADS

## Abstract

**Purpose:**

Non-invasive follicular thyroid neoplasm with papillary-like nuclear features (NIFTP) is a premalignant tumor formerly known as non-invasive encapsulated follicular variant of papillary thyroid carcinoma. The aim of this study was to investigate the clinicopathological traits of NIFTP to discern them from well-differentiated cancers and potentially avoid overtreatment.

**Methods:**

We conducted a retrospective cohort study of NIFTP cases from July 2017 to November 2022 at our center. A review of demographic, clinical, sonographic, cytologic and surgical data was performed.

**Results:**

During the study period 70 NIFTP cases were included. Among the cohort 74.3% (52/70) were women and the mean age was 55 years (range, 25–84). The majority of patients presented euthyroid (92.9%). Median NIFTP size was 2.5 cm (range, 1.0-10.8). Most nodules displayed low or intermediate risk on ultrasound being labeled EU-TIRADS 3 (46/70, 65.7%) and EU-TIRADS 4 (23/70, 32.9%). In cytology they were typically diagnosed as Bethesda III (15/70, 21.4%) or Bethesda IV (41/70, 58.6%). Regarding surgical procedures, 36 patients (51.4%) underwent lobo-isthmectomy and 34 patients (48.6%) received total thyroidectomy. Thirteen patients (18.6%) had coexisting microcarcinomas. No patients received radioiodine ablation. After a median follow-up of 27.5 months, no structural or biochemical recurrences were observed.

**Conclusion:**

Non-suspect thyroid nodules on preoperative ultrasound when combined with an indeterminate cytology and altered molecular profile should raise awareness towards the possibility of NIFTP. Even though neither FNAB nor CNB are definitive for NIFTP, CNB may be considered when additional architectural assessment is needed. Management with lobectomy seems to suffice unless total thyroidectomy is justified.

## Introduction

The concept of non-invasive follicular thyroid neoplasm with papillary-like nuclear features (NIFTP) was first coined in 2016, as a reclassified subset of non-invasive encapsulated follicular variant of papillary thyroid carcinoma [1]. It has been subsequently acknowledged in the fourth edition of the WHO Classification of Tumours of Endocrine Organs, being a compromise term to avoid the word carcinoma, once NIFTP is considered a borderline lesion with very low malignant potential and overall indolent behavior [2]. Therefore, the nomenclature change was proposed to improve patient care by preventing overdiagnosis, destigmatizing malignancy, and ideally scale down treatment and follow-up [3].

The initially predicted rate of NIFTP among papillary thyroid carcinoma (PTC) cases was overestimated by Nikiforov et al. as a following meta-analysis revealed NIFTP prevalence of 9.1% in all PTC cases [1, 4]. A lower NIFTP incidence has been observed in Asia compared to Western practice and attributed to interobserver variation of pathologic approaches, more than ethnic disparities [4, 5]. Similarly to other thyroid neoplasms, NIFTP is female-predominant (gender ratio of 3:1 to 4:1), with patients usually presenting from fourth to sixth decades of life [1, 6, 7].

NIFTP is only diagnosable postoperatively through histological examination because the capsule cannot be evaluated by fine-needle aspiration biopsy (FNAB), whereas frozen sections are not helpful either [8]. Histology allows to reliably distinguish NIFTP from invasive follicular variant PTC (FVPTC) which exhibits identical follicular growth pattern and nuclear features [3, 9, 10]. Although the definite diagnosis requires surgical excision, preoperative suspicion should be raised towards nodules which could expectedly correspond to NIFTP. Knowledge of clinical presentation, ultrasound features, cytological characteristics and molecular profile of these tumors is trivial to define the extent of surgery, given that a more conservative management with lobo-isthmectomy is favorable [11, 12].

The aim of this study was to investigate the clinicopathological traits of NIFTP, to tell them from well-differentiated cancers and potentially avoid overtreatment.

## Materials and methods

We conducted a retrospective cohort study of consecutive cases diagnosed as NIFTP from July 2017 to November 2022 identified through electronic medical records at our tertiary care center. A review of demographic, clinical, sonographic, cytologic and surgical data was performed. Ultrasound assessment of thyroid nodules and stratification of requirement for FNAB went according to the European Thyroid Imaging and Reporting Data System (EU-TIRADS) as disclosed in Table 1 [12]. Exclusion criteria encompassed pathology consultation cases for other institutions, multinodular goiter patients in which NIFTP were not preoperatively submitted to FNAB, and NIFTP with a diameter ≤ 1 cm (microNIFTP). As for the latter, EU-TIRADS guidelines discourage FNAB for subcentimetric nodules provided that there are no abnormal lymph nodes, prompting diagnosis of such cases as supplementary findings after surgery for a different motive in our practice. Written informed consent was obtained from the patients to use their clinical and biochemical data for research purposes. Statistical analysis was executed with SPSS Statistics (version 29, Armonk, NY: IBM Corp).

**Table 1:** European thyroid imaging and reporting data system (EU-TIRADS)

Category	Ultrasound features	Malignancy risk (%)	Recommendations
EU-TIRADS 1: normal	No nodules	None	None
EU-TIRADS 2: benign	Pure cyst Entirely spongiform	≅ 0	No FNAB unless compressive
EU-TIRADS 3: low risk	Ovoid, smooth isoechoic/hyperechoic No features of high suspicion	2–4	FNAB if > 20 mm
EU-TIRADS 4: intermediate risk	Ovoid, smooth, mildly hypoechoic No features of high suspicion	6–17	FNAB if > 15 mm
EU-TIRADS 5: high risk	At least 1 of the following features of high suspicion: – Irregular shape – Irregular margins – Microcalcifications – Marked hypoechogenicity (and solid)	26–87	Active surveillance FNAB if > 10 mm

Modified from Russ G, Steen M, Durante C, Ngu R, Leenhardt L (2017) European Thyroid Association Guidelines for Ultrasound Malignancy Risk Stratification of Thyroid Nodules in Adults: The EU-TIRADS. European Thyroid Journal 6(5): 225–237 [13]

## Results

**Fig. 1:**
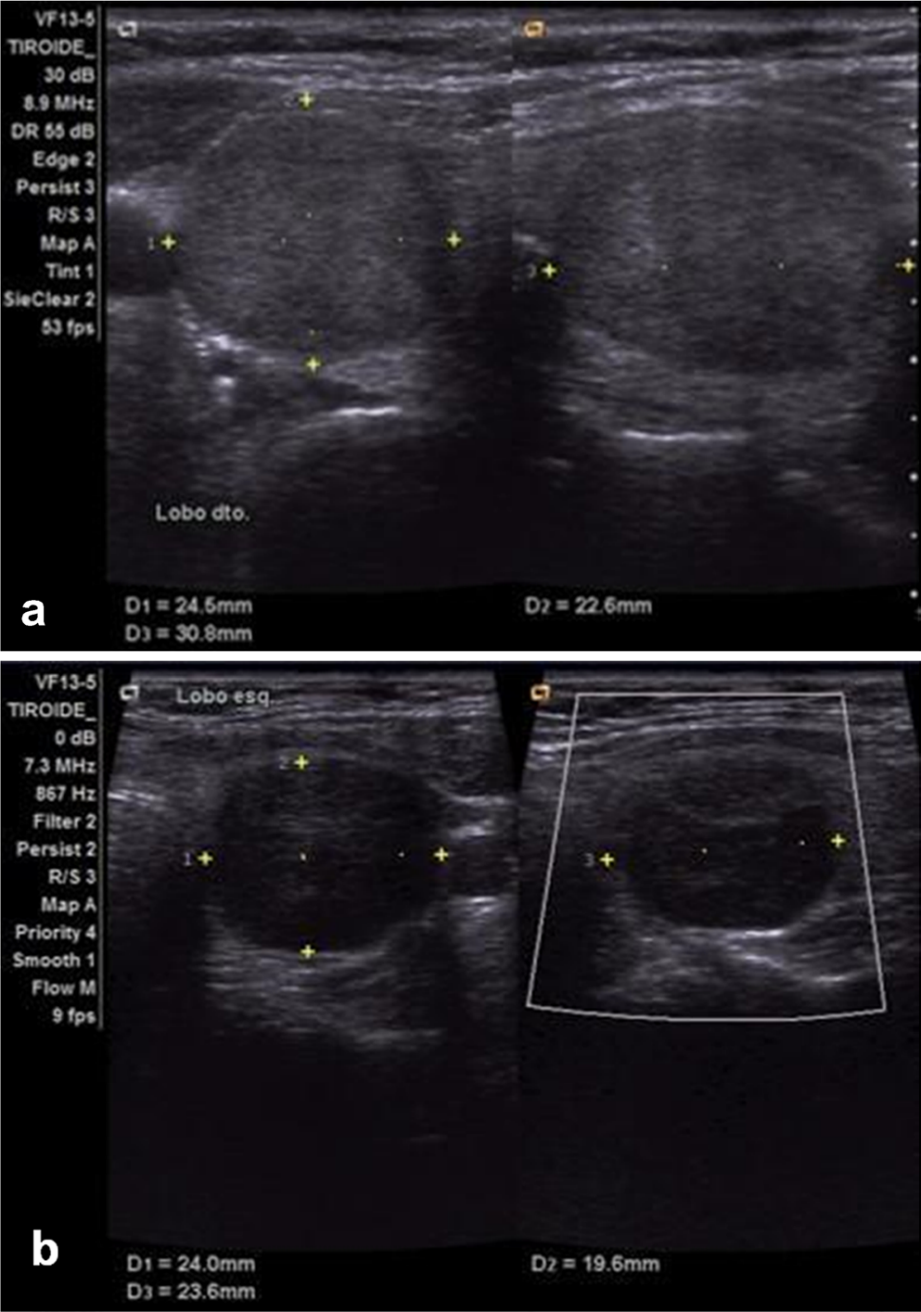
(**a**) An isoechoic, ovoid, and smooth right-sided thyroid nodule with a thin halo measuring 25 × 23 × 31 mm – EU-TIRADS 3. (**b**) A mildly hypoechoic, ovoid, and smooth left-sided thyroid nodule with absent internal or peripheral flow measuring 24 × 20 × 24 mm – EU-TIRADS 4

**Fig. 2:**
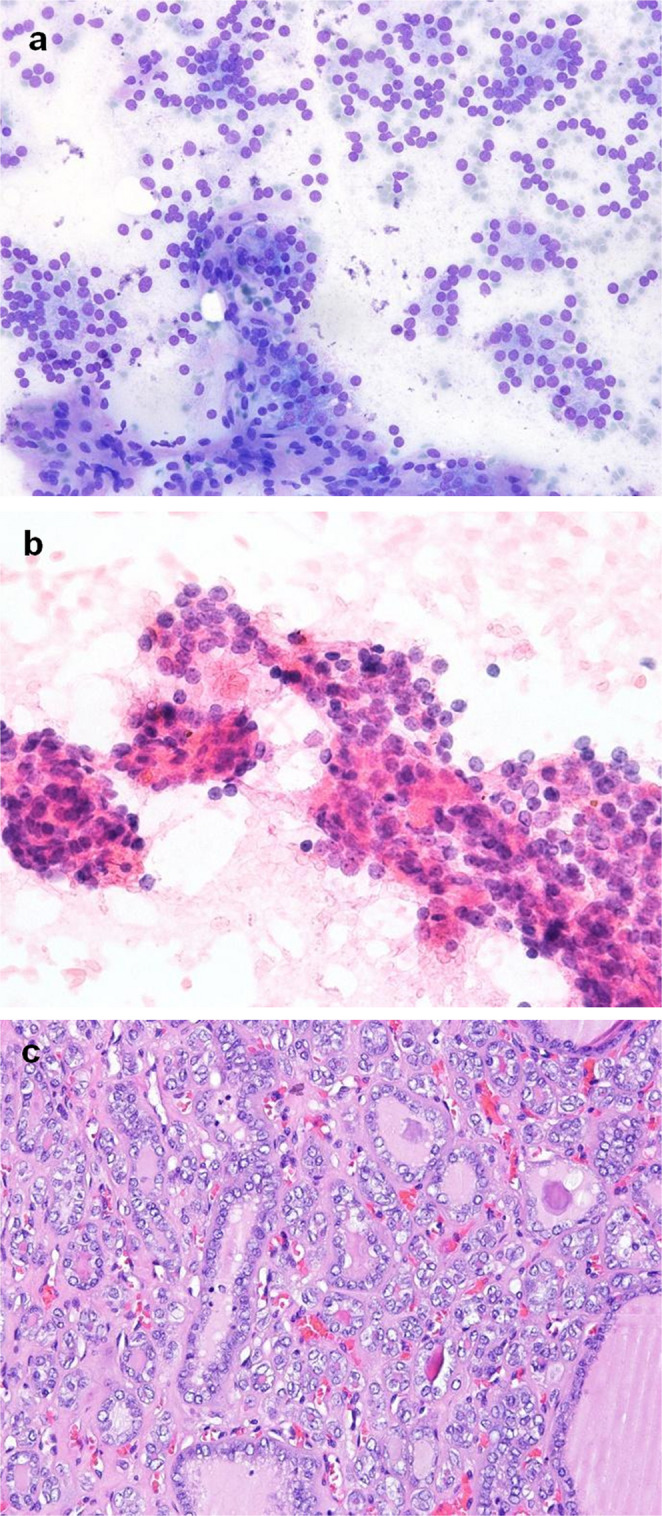
(**a**) Follicular neoplasm - Follicular cells arranged in microfollicles and some crowded clusters. On the histological specimen this lesion was diagnosed as NIFTP. [May-Grünwald giemsa stain, 200x]. (**b**) Atypia of Undetermined Significance – nuclear and architectural atypia. Crowded groups entrapped in some fibrin strands, with some cells with irregular nuclear membranes and grooves. On the histological specimen this lesion was diagnosed as NIFTP. [hematoxylin and eosin stain, 400x]. (**c**) NIFTP – Tumor with a follicular growth pattern composed of cells with enlarged nuclei, irregular nuclear membranes with frequent grooves and clearing chromatin (score 3). [hematoxylin and eosin stain, 200x]

During the study period, 121 patients were initially identified and 70 NIFTP cases were included. Among the cohort, 74.3% (52/70) were women and the mean age was 55 years (range, 25–84). In respect of thyroid function, the vast majority of patients presented euthyroid (92.9%). Median NIFTP size was 2.5 cm (range, 1.0–10.8). Most nodules displayed low or intermediate risk sonographically being labeled EU-TIRADS 3 (46/70, 65.7%) and EU-TIRADS 4 (23/70, 32.9%) – see Fig. 1. On cytology these typically matched indeterminate categories in the Bethesda System for Reporting Thyroid Cytopathology (TBSRTC) as atypia of undetermined significance (AUS) - Bethesda III (15/70, 21.4%) or follicular neoplasm (FN) - Bethesda IV (41/70, 58.6%). Figure 2 shows cytological smears of nodules obtained by FNAB and the histologic appearance of a NIFTP specimen. Regarding surgical procedures, 36 patients (51.4%) underwent lobo-isthmectomy and 34 patients (48.6%) received total thyroidectomy. There were no cases of completion thyroidectomy. Concerning postoperative complications, temporary hypoparathyroidism (hypoPT) occurred in 5 cases (7.1%), while 3 patients (4.3%) suffered transient recurrent laryngeal nerve (RLN) palsy after total thyroidectomy. Thirteen patients (18.6%) had coexisting microcarcinomas. None of the patients received radioiodine ablation and thyrotropin-stimulating-hormone (TSH) levels were kept between 0.5 and 2 mIU/L upon multidisciplinary decision. After a median follow-up of 27.5 months no structural or biochemical recurrences were observed. A detailed overview of NIFTP preoperative characteristics and surgical outcomes is exhibited in Table 2.

**Table 2:** Preoperative data and patient outcomes sorted by surgical procedure

	Lobo-isthmectomy (*n* = 36)	Total thyroidectomy (*n* = 34)	Total (*n* = 70)
**Age** (years), mean (range)	53 (26–84)	57 (25–84)	55 (25–84)
**Gender**			
Female (n, %)	24 (34.3)	28 (40.0)	52 (74.3)
Male (n, %)	12 (17.1)	6 (8.6)	18 (25.7)
**Family history of thyroid cancer** (n, %)	5 (7.1)	2 (2.9)	7 (10.0)
**Thyroid function** (n, %)			
Hypothyroid	1 (1.4)	2 (2.9)	3 (4.3)
Euthyroid	35 (50.0)	30 (42.9)	65 (92.9)
Hyperthyroid	-	2 (2.9)	2 (2.9)
**Tumor size** (mm), median (range)	21 (11–48)	31 (10–105)	25 (10–105)
**Echogenicity** (n, %)			
Hypoechoic	14 (20.0)	8 (11.4)	22 (31.4)
Isoechoic	19 (27.1)	26 (37.1)	45 (64.3)
Hyperechoic	3 (4.3)	-	3 (4.3)
**Composition** (n, %)			
Solid	29 (41.4)	23 (32.9)	52 (74.3)
Mixed	7 (10.0)	11 (15.7)	18 (25.7)
**EU-TIRADS** (n, %)			
3	20 (28.6)	26 (37.1)	46 (65.5)
4	15 (21.4)	8 (11.4)	23 (32.9)
5	1 (1.4)	-	1 (1.4)
**Bethesda** (n, %)			
I	2 (2.9)	-	2 (2.9)
II	4 (5.7)	8 (11.4)	12 (17.1)
III	6 (8.6)	9 (12.9)	15 (21.4)
IV	24 (34.3)	17 (24.3)	41 (58.6)
**Associated microcarcinoma** (n, %)	3 (4.3)	10 (14.3)	13 (18.6)
**Temporary post-op hypoPT** (n, %)	-	5 (7.1)	5 (7.1)
**Transient RLN palsy** (n, %)	-	3 (4.3)	3 (4.3)
**Follow-up** (months), median (range)	29.5 (4–65)	27.0 (2–61)	27.5 (2–65)

## Discussion

NIFTP is a fairly recently established entity whose diagnosis must be strongly supported by strict criteria. These include encapsulation or clear demarcation of the tumor, follicular growth pattern and nuclear score ≥ 2 [1, 6, 12, 14]. A 3-point grading system of nuclear features of papillary carcinoma assigns minimal ‘atypia’ value of 2 to render NIFTP diagnosis in place of follicular adenoma or adenomatous nodule [3, 7, 15]. Furthermore, the absence of exclusion criteria is imperative, i.e.: vascular or capsular invasion; ≥1% papillae; any psammoma bodies; >30% solid/trabecular/insular growth pattern; tumor necrosis or high mitotic activity; microscopic features of other specific variants of papillary carcinoma [1]. In fact, the originally accepted rule of ‘<1% papillae’ was challenged by regional lymph node micrometastasis reports in tumors that assumingly met NIFTP requirements, motivating a change of criteria to ‘no well-formed papillae’ in the 2018 consensus group revision [16]. Afterwards, several papers revealed absence of metastases and recurrences in cases with ≤ 1% papillae, as much as a Memorial Sloan Kettering Cancer Center study stating that lymph node metastases were only observed in patients ≥ 10% papillae [17]. Ultimately, the 2022 WHO Classification reinstated the original criteria allowing < 1% papillae, and highlighted the importance to distinguish between true papillae and pseudopapillary structures [18].

Clinical presentation of NIFTP is non-specific since these are typically asymptomatic nodules which get detected during physical examination or incidentally in diagnostic neck imaging, as a single nodule, or a lesion among multinodular background instead [11]. Though seldom occurring, sizable growth might cause mass effect on adjacent structures leading to compressive symptoms such as dysphonia, globus sensation or airway compromise [15, 19]. About thyroid function, patients most often remain euthyroid according to literature and our results are concurring (92.9%) [20, 21]. It was beyond the scope of this study to actively search for given symptoms that could indicate NIFTP, which are rather uncertain, while our main focus relied on correlating preoperative ultrasound and cytologic features.

On the topic of sonography, NIFTP lacks malignant ultrasound features being more similar to benign nodular disease like follicular adenoma [22]. Yang et al. reclassified 179 cases of FVPTC resulting in 72 cases (40.2%) of NIFTP [7]. After revision, the ultrasound for infiltrative FVPTC group showed at least one worrisome characteristic: marked hypoechogenicity, ‘taller than wide’ shape, blurred margins, microcalcifications or avascular color doppler. On the other hand, NIFTP presented as well-circumscribed oval/round nodules with a hypoechoic rim. In a retrospective study of 208 patients Hahn et al. concluded that NIFTP had more hyper or isoechogenicity (*p* = 0.043) and no microcalcifications (*p* = 0.031) compared to non-NIFTP, resulting in a considerably lower rate of high suspicion for malignancy (14.7% vs. 37.9%, *p* = 0.024) [23]. In addition, surgical indication for NIFTP was significantly better suggested by core needle biopsy (CNB) in comparison to FNAB (100% vs. 54.3%, *p =* 0.008). Authors argue that a modified CNB technique containing the nodule, a capsular fragment, and surrounding parenchyma is valuable in reducing sampling error and increasing diagnostic yields [24]. Among our cohort, although hyperechogenicity was not very expressive, almost two-thirds of NIFTP nodules (45/70, 64.3%) were isoechogenic and the remainder slightly hypoechogenic (22/70, 31.4%). Our results point out that on ultrasound NIFTP are commonly classified as low or intermediate risk (EU-TIRADS 3 and 4) what is in line with comparable studies [22, 23, 25].

Risk of malignancy (ROM) plays a pivotal role in TBSRTC, thoroughly influencing consequent clinical decisions [26]. The adoption of NIFTP terminology, bearing its non-malignant nature, has resulted in a significant decrease in ROM, particularly on indeterminate categories of TBSRTC to which NIFTP nodules often correlate [3, 8, 27]. In a large multicentric study of 6943 thyroid nodules by Faquin et al., ROM had a decrease of 5.2% in AUS – Bethesda III, 9.9% in FN – Bethesda IV, and 17.6% in ‘suspicious for malignancy’ (SM) – Bethesda V (*p* < 0.05) [28]. Despite the fact that our cytologic results were mainly fitting of indeterminate categories, similarly to articles by both Ruanpeng et al. and Bongiovanni et al., we comparatively noticed a lower proportion of AUS (21.4%), a higher ratio of FN (58.6%), and oddly no SM cases [29, 30].

Even though preoperative molecular analysis of NIFTP nodules does not concede a definitive diagnosis, it may be useful in differentiation from classic PTC or other PTC variants [11]. The molecular profile of NIFTP holds no signature marker, sharing genetic abnormalities with other follicular-patterned thyroid tumors instead [31]. NIFTP predominantly harbors activating *RAS* mutations in 30–67% of cases, particularly *NRAS*, but also *HRAS* and infrequently *KRAS* [19, 27]. Rearrangements in *PAX8-PPARG* and *THADA* fusions have also been found in a minor subgroup of patients [9, 15]. Notwithstanding that rare *BRAFK601E* mutations might traduce a better outcome, it must be noted that high-risk mutations such as *BRAF600E*, *TERT* promoter or TP53 disqualify a tumor from being classified as NIFTP, thus warranting additional search for eventual invasive features or hidden papillae [18, 19]. While Afirma^®^ Gene Expression Classifier (Afirma GEC) and ThyroSeq^®^ are renowned and effective molecular evaluation systems for thyroid nodules, it is not our current practice to perform preoperative testing owing to financial cost, what poses a restraint on this study [11].

According to the American Thyroid Association’s position paper, NIFTP should be managed under the same algorithm of low-risk differentiated thyroid cancer (DTC) as per 2015 Thyroid Nodule and DTC guidelines [32]. These recommendations reckon lobectomy as sufficient and the preferred procedure, advising against unnecessary remnant ablation. Postoperatively, maintenance of TSH target levels within the range of 0.5–2 mIU/L is encouraged [33]. The type and extent of surgical treatment ought to rely on a multimodal assessment consisting of physical examination, ultrasound features, cytologic characteristics, and ideally molecular profiling data if available [19]. Good judgment in the necessary amount of surgery needs to take into account the presence of bilateral disease and multifocal disease, making total thyroidectomy a viable option for some NIFTP cases [12]. When considering a lobectomy, patients should be alerted about the prospect of an extra surgery for completion thyroidectomy [27]. Although to the best of our knowledge there are currently no guidelines on follow-up after NIFTP resection, surveillance with periodic serum thyroglobulin (Tg) measurements and neck ultrasound imaging is recommended, depending upon individual context [15, 31]. Non-existent anti-Tg antibodies and normal neck sonography findings sustain a low-risk status, what may ease the frequency of routine assessments [12].

Literature review apart, this is a descriptive study missing formal statistical comparison of data between concurrent NIFTP and PTC cases, striking as a limitation. Moreover, the moderately short follow-up does not allow us to exclude eventual recurrences.

## Conclusion

The recognition of NIFTP has shifted the diagnostic and therapeutic approach for a sector of premalignant thyroid nodules, reducing the likelihood of overtreatment. Besides, a mostly semantic change helped alleviate the psychosocial impact associated with cancer. A low or intermediate risk on ultrasound, accompanied by indeterminate cytology, and ideally the presence of altered preoperative molecular profile, should raise suspicion of NIFTP, thus leading surgeons to ponder less aggressive surgical treatment. Although not definitive for NIFTP, CNB may offer better preoperative risk stratification as it provides architectural information and higher diagnostic yield for follicular-patterned lesions in comparison to FNAB, possibly reducing indeterminate rates. Evidence-based literature on the long-term follow-up of these tumors is required, to further prove favorable prognosis, and validate current management strategies.

## Data Availability

No datasets were generated or analysed during the current study.
